# Point-prevalence survey of antimicrobial use in intensive care units in Nepal

**DOI:** 10.1017/ash.2024.83

**Published:** 2024-05-08

**Authors:** Sabin Koirala, Agnimshwor Dahal, Hem Raj Paneru, Astha Thapa, Surendra Bhusal, Roshni Shakya, Subekshya Luitel, Kanchan Koirala, Diptesh Aryal

**Affiliations:** 1 Hospital for Advanced Medicine and Surgery (HAMS), Kathmandu, Nepal; 2 Nepal Intensive Care Research Foundation, Kathmandu, Nepal; 3 John H. Stroger Jr. Cook County Hospital, Chicago, IL, USA; 4 National Academy of Medical Sciences (NAMS), Bir Hospital, Kathmandu, Nepal

## Abstract

**Objective:**

This study aimed to investigate the prevalence and practices of antibiotic use in intensive care units (ICUs) in Nepal and to identify potential areas for implementing antimicrobial stewardship programs.

**Design:**

A point prevalence survey was conducted to characterize and quantify the antimicrobial utilization in level III ICUs of Nepal.

**Methods:**

Data on antibiotic prescription rates, reasons for prescribing antibiotics, and prescribing practices were collected and analyzed. The prevalence of antimicrobial resistance was also assessed.

**Results:**

The antibiotic prescribing rate was found to be very high, with 92.85% of patients in ICU on antibiotics. Prolonged surgical prophylaxis was the most common reason for prescribing antibiotics. Empirical therapy accounted for 67.5% of all antibiotic prescriptions. Prescribing practices were poor, with low adherence to guidelines and best practices. Broad-spectrum antibiotics were commonly used even for surgical prophylaxis or community-acquired infections. High resistance was observed against commonly used antibiotics.

**Conclusions:**

The study underscores the urgent need for effective antimicrobial stewardship programs in ICUs of Nepal. Implementing robust stewardship programs could help optimize antibiotic utilization, improve patient outcomes, and combat the global threat of antimicrobial resistance. The findings serve as a stepping stone toward understanding and improving antibiotic prescribing practices in ICUs of Nepal.

## Introduction

Since the discovery of antimicrobials in the late 1920s, they have been seen as a miracle treatment for infectious diseases and their usage has grown tremendously.^1^ Since then antimicrobial resistance (AMR) has slowly and steadily emerged and progressed to the point where infection with AMR has led to serious illnesses, prolonged hospital admissions, increased healthcare costs, higher costs in second-line drugs, and treatment failures.^2^ The situation has inflated to the point where antimicrobials may be causing more harm than good.^3^

The global problem of AMR is particularly pressing in developing countries, where the infectious disease burden is high and cost constraints prevent the widespread application of newer, more expensive agents.^4^ In these countries AMR is driven by the high incidence of infectious diseases,^2^ inappropriate use of antimicrobials in treatment,^5^ use of antimicrobials as growth promoters^6,^ and lack or poor implementation of legislation to AMR.^7^ AMR is, nevertheless, a global issue of public health concern.

Infection is the most common presentation among hospitalized patients in intensive care unit (ICU), and in many instances, is a determining factor for patient outcomes.^8,9^ Antimicrobials have become an essential part of treatment in all spectrums of critically ill patients. Stronger antimicrobials are particularly used in critical care.^7^ This may have escalated antimicrobial resistance to a newer dimension. Hence, ICUs are now often recognized as the epicenter of infections in the hospital.^10^

The situation in Nepal is comparable. There has been an increasing burden of antimicrobial resistance (AMR) in Nepal over the last two decades.^11–15^ However, there is a lack of data on antimicrobial use in ICUs of Nepal. This data is important to help develop pertinent strategies to improve the future use of antimicrobials in hospitals as part of the National Action Plan for Containment of Antimicrobial Resistance 2016.^16^ We intend to conduct a point prevalence survey to characterize and quantify the antimicrobial utilization in level III ICUs of Nepal. This study will inform, at the policy level, the strategies to implement antimicrobial stewardship programs and develop antimicrobial guidelines in Nepal.

## Methods

### Study design and setting

A multicentre quantitative cross-sectional study was conducted in various ICUs in Nepal. The study was conducted for one month after getting ethical approval from Nepal Health Research Council. WHO methodology for PPS and the global point prevalence survey method were adopted, which are standardized methods for surveillance of antibiotic use and validated tools to assess the quality of antibiotic prescribing. Antibiotics studied in this study were classified according to the Anatomic Therapeutic Chemical (ATC) methodology developed by the WHO Collaborating Center for Drug Statistics Methodology in Oslo, Norway. Only antibiotics listed in Annex XI of the referred document and administered through oral, parenteral, rectal, or inhalation routes were included in the survey. For example, topical applications, eye drops, ear drops, and vaginal suppositories were excluded.

### Participants

As per WHO methodology and Global-PPS form, all the adult patients (>18 yr) admitted to the participating ICUs as an inpatient at or before 08:00 were included in the survey irrespective of whether they were receiving antibiotic treatment or not. All inpatients admitted in the ICU at 8 o’clock in the morning on the day of survey were counted in the denominator. All inpatients “on antibiotic agents” at 8 o’clock in the morning on the day of survey were included in the numerator. All daycare hospitalizations, all patients admitted after 8 a.m. on the day of the survey, and all patients who received surgical prophylaxis after 8 a.m. on the day of the PPS were excluded. Definition of “on antibiotic agents”: A patient receiving an antibiotic eg every 48 hours but not receiving this antibiotic on the survey day must be included = ongoing antibiotic treatment. An antibiotic prescribed at one o’clock (during the ward round or when results become available or for surgical prophylaxis) in the afternoon on the day of the survey must not be included (not active or ongoing at 8 o’clock in the morning). For patients receiving surgical prophylaxis, the administration of antibiotic prophylaxis should be checked in the previous 24 hours in order to encode the duration of prophylaxis as either one dose, one day (=multiple doses given in one day), or >1 day. This means that patients who received the surgical prophylaxis ‘before’ 8 a.m. on the day of the PPS will be included in the survey.

### Sample size determination and sampling technique

The sampling technique recommended by WHO Methodology for Point Prevalence Survey on Antimicrobial Use in Hospitals Version 1.1 was utilized as follows: All the ICU patients fulfilling the inclusion criteria were included in the studies from all the involved hospitals.

Convenience sampling techniques were used for both hospitals and patient-level sampling. The sampling was done in each ICU on the day of the survey. The data collector prepared a list of all eligible patients according to the inclusion criteria. The list was ordered alphabetically according to patients’ surnames (not by bed or patient number).

### Data collection and management

Data was collected using three Google forms, one for hospital level and two ward-level and one for patient-level data, as described in the Global-PPS method. The principal source for completing data collection was through a review of the patient’s medical records. The treating physician was interviewed when needed for clarification. Patient’s prescriptions and files were accessed at 2.00 p.m.. However, information was collected only up to 8:00 a.m. in the morning of the same day and further changes beyond that time were excluded.

Before initiating the full survey, a pilot study was also conducted in one of the participating hospitals by, for example, reviewing clinical notes for up to 10 patients involving the whole investigator team.

Data collected after surveying each hospital were sorted and organized to prevent mix-up during data entry. All the data collected in the study were entered into a Google Form and exported into MS-Excel/SPSS for analyses.

## Results

### Demographic characteristics of patients and participating hospitals

Total of 98 patients, from 12 ICUs of 11 hospitals, were enrolled in the study. Of all the patients included in the study, the majority (54.08%) were from private-for-profit institutes. Among these patients, 63 (64.28%) were undergoing treatment for a medical cause (Table 1).

**Table 1.** Patient and hospital characteristics

Variable		N	Mean ± SD OR %
Patient age	13–39 yrs	28	53.66 ± 20.92
	40–64 yrs	38	
	>65 yrs	32	
Gender	Male	57	58.16%
	Female	41	41.83%
Type of treatment	Medical	63	64.28%
	Surgical	35	35.71%
Antibiotics use	On Antibiotics	91	92.85%
	NOT on Antibiotics	7	7.14%
Hospital ownership	Public	45	45.92%
	Private not for profit	–	–
	Private for profit	53	54.08%

### Antibiotics consumption

**Figure 1.**
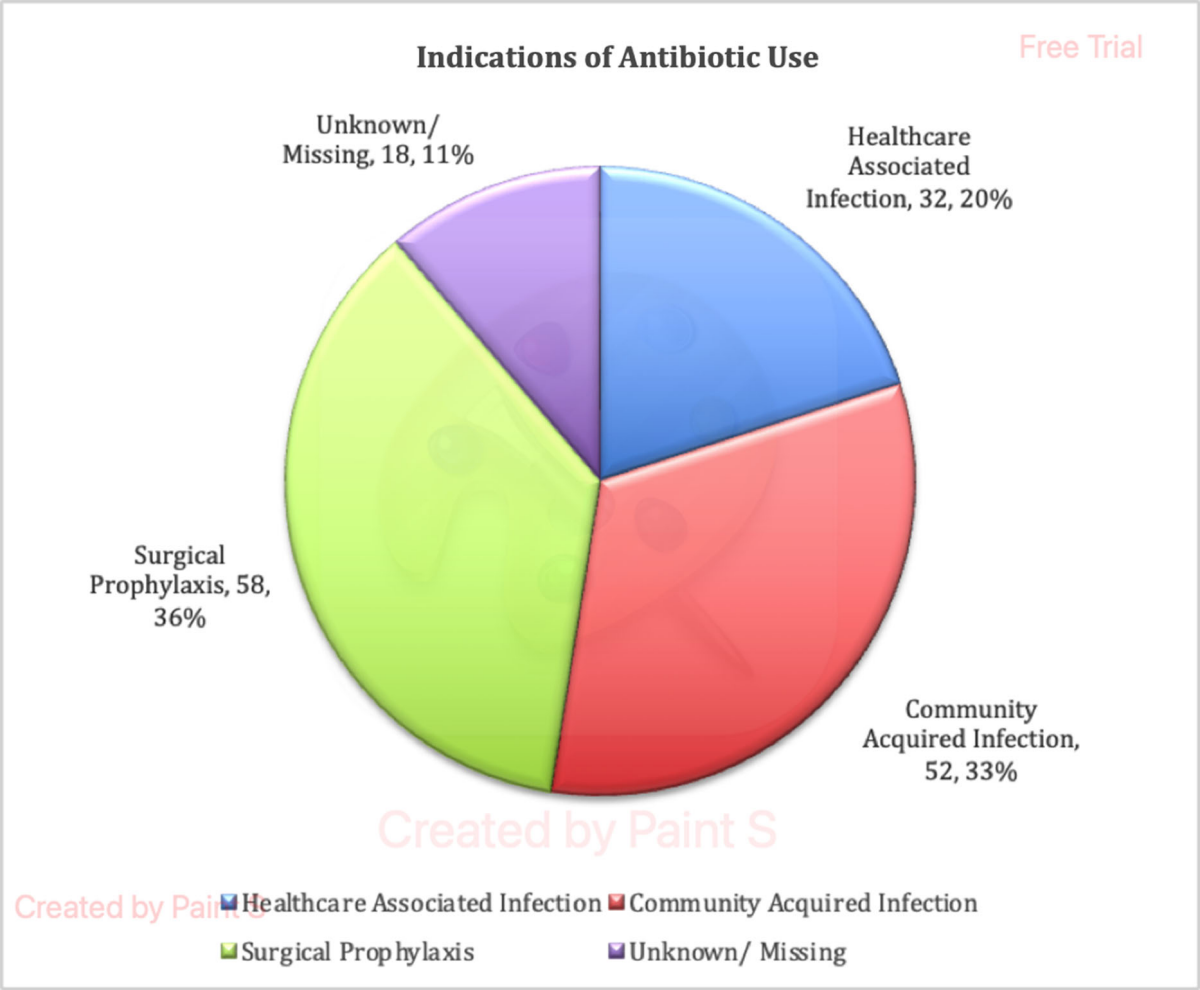
Indications of antibiotic use.

In total, 91 (92.85%) patients were on at least one antibiotic at the time of the PPS. 56 patients (34.78%) were on two antibiotics, 13 patients (8.07%) were on three antibiotics and one patient (0.62%) was on more than 3 antibiotics. Total number of antibiotics received by all 98 patients combined was 160. The most common indication for the use of antibiotics was surgical prophylaxis (36.25%), followed by community-acquired infection (32.50%) and healthcare-associated infection (20%) (Figure 1).

**Figure 2.**
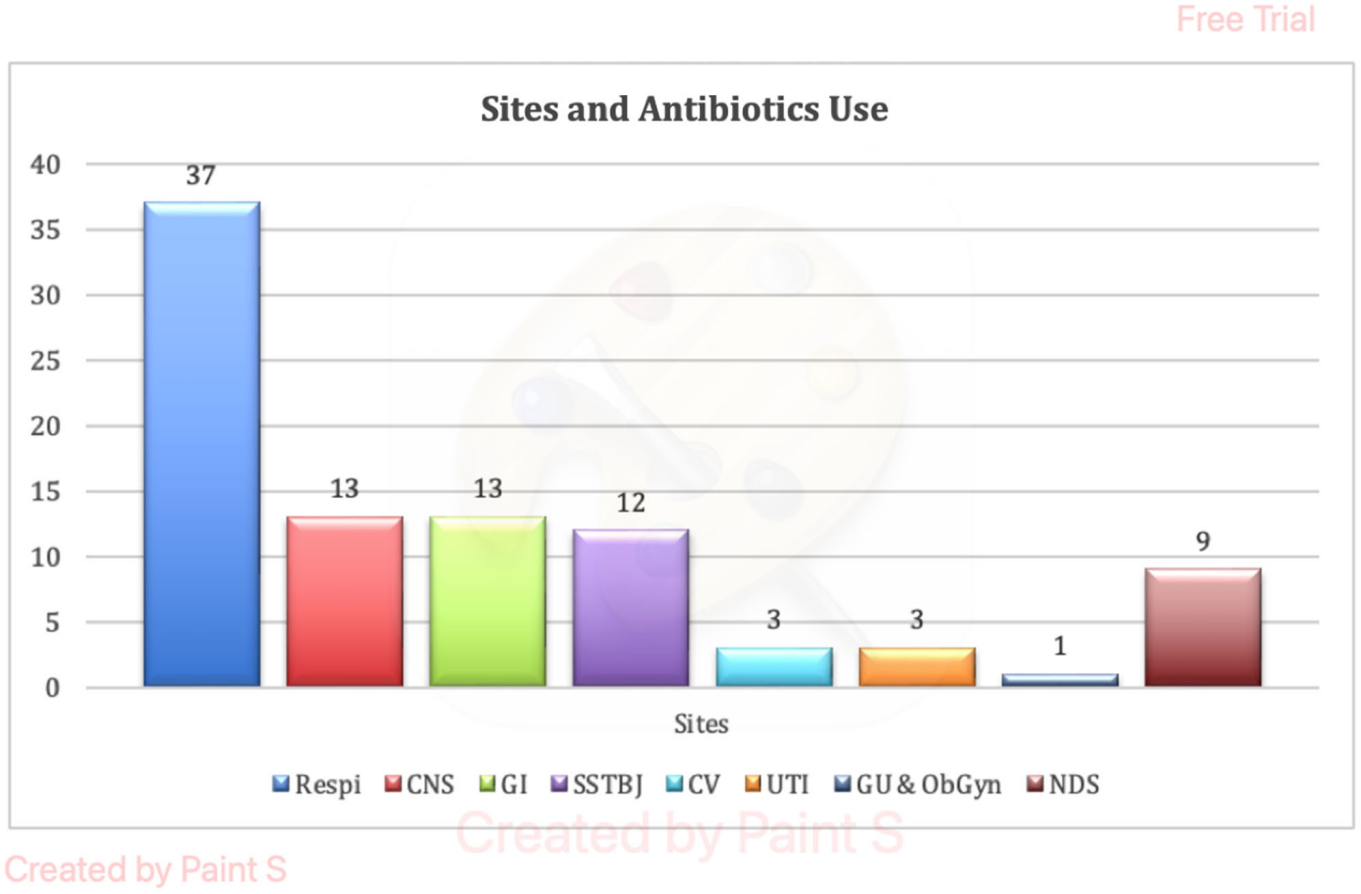
Sites and antibiotic use.

Respiratory system was the commonest body system (40.70%) for which antibiotics were being prescribed and this was followed by Central Nervous System (CNS) (14.30%), Gastrointestinal system (14.30%), and Skin, soft tissue, bone, and joint (13.2 %) (Figure 2).

**Figure 3.**
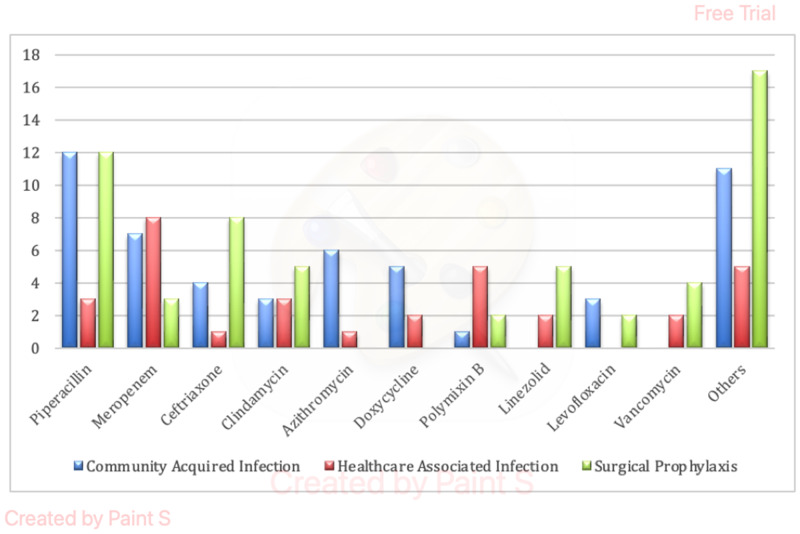
Antibiotics usage by drug.

Piperacillin was the most commonly used antibiotic and was used in 35.16% of all patients receiving antibiotics. This was closely followed by Meropenem (22.2%) and Ceftriaxone (17.8%). Polymyxin B was prescribed in 8.9%, Linezolid in 7.8% and Vancomycin was used in 6.7% of all patients receiving antibiotics. Piperacillin was the most commonly used antibiotic for both community-acquired infections and surgical prophylaxis, while Meropenem was the most commonly used antibiotic for hospital-acquired infections (Figure 3).

### Antibiotics prescribing practice

Of all the antibiotic prescriptions (160), 108 (67.5%) were empirical therapy and 49 (30.6%) were targeted (3 patients had missing data). Of all these prescriptions, most didn’t have a stop or review date mentioned nor were compliant with local guidelines. Use of biomarkers to start or change antibiotics was low (35.16%) (Table 2).

**Table 2.** Prescribing practice

Practice	N/Total (%)
Stop/review date recorded	23/160 (14.37%)^a^
Use of biomarkers to start or change Antibiotics	32/91 (35.16%)
Compliant to local guidelines	44/160 (27.50%)^a^
Cultures sent to document Infection	56/91 (61.53 %)
Cultures sent prior to starting or changing Antibiotics	31/160 (34.4%)

a Total number of antibiotics received by all 98 patients combined was 160.

### Microbiology

Of all the patients on antibiotics (91), cultures were sent to 56 patients (61.53%). Among these 56 patients, cultures were sent before starting antibiotics in 31 patients (55.36%). Total of 112 samples were sent for culture. Majority of the samples were Blood (35), Sputum/bronchial aspirate (35), and Urine (34).

Of all the cultures sent, the majority (56.25%) were negative for any growth, and 26.78% of the samples grew an organism (Table 3). Among the cultured organisms, Klebsiella pneumoniae was the most common, appearing in 5 instances across various samples. Citrobacter freundii followed closely with 4 occurrences. Escherichia coli and Staphylococcus aureus were each found 3 times. Notably, 12 of the cultured organisms (40%) were isolated from sputum/ bronchial aspirate samples.

**Table 3.** Microbiological diagnosis

Variable	N (%)
Sample collected for microbiological workup	
Yes	56/91
No	35/91
Samples sent before starting/changing antibiotics	
Yes	31/56
No	25/56
Specimen type	
Blood	35/112
Sputum/bronchial aspirate	35/112
CSF	3/112
Urine	34/112
BAL	1/112
Wound	4/112
Other	–
Culture results	
Positive	30/112 (26.78%)
Negative	63/112 (56.25%)
Awaited/unknown	19/112 (16.96%)

### Antibiotics resistance

**Figure 4.**
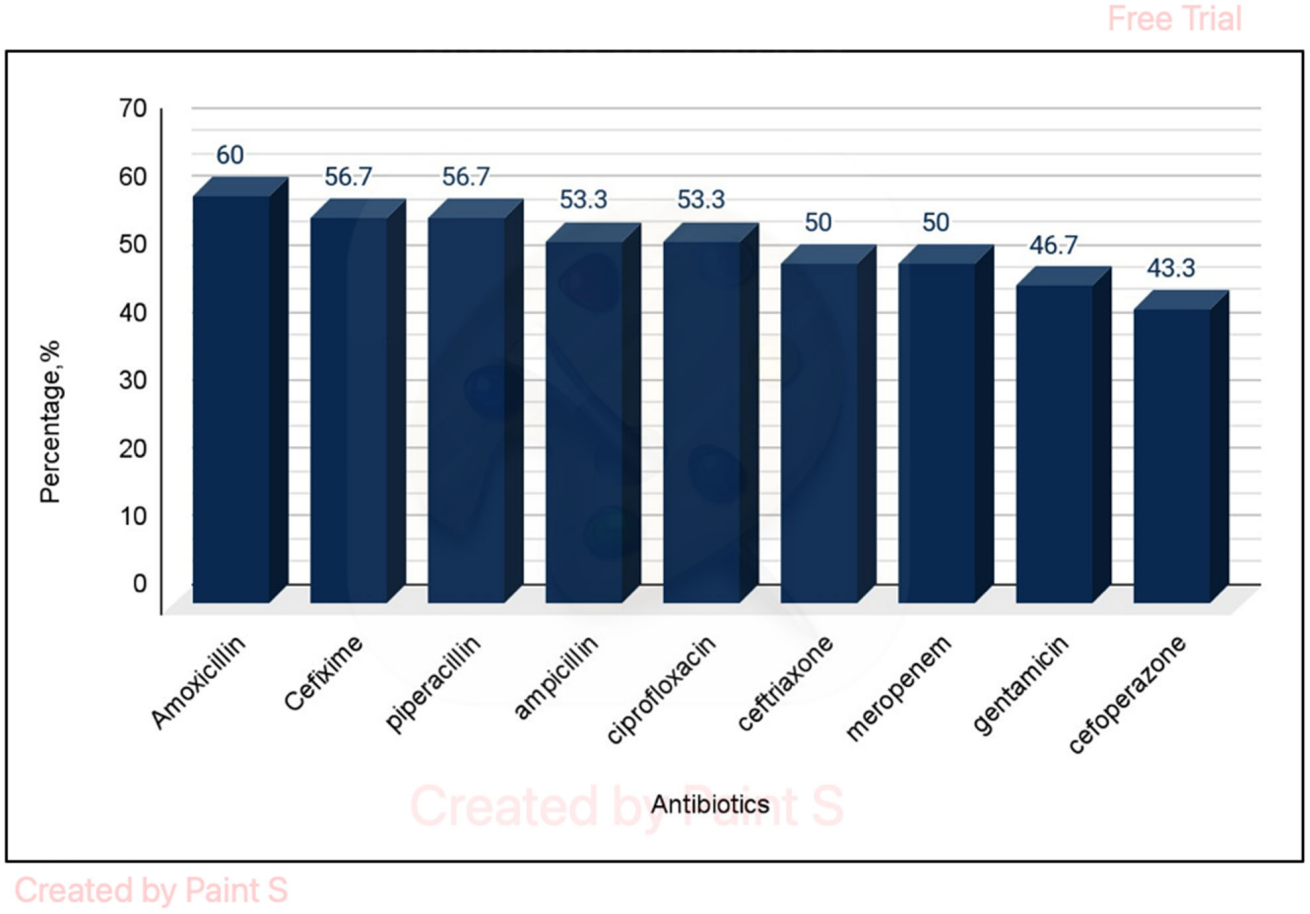
Antibiotics resistance.

Piperacillin was the most commonly used antibiotic (35.6%), followed by Meropenem (22.2%) and Ceftriaxone (17.8%). It was observed that 60% of the positive cultures exhibited resistance to Amoxicillin, while 50% of the positive cultures showed resistance to Carbapenem. (Figure 4).

## Discussion

Antimicrobial resistance is a global issue and the burden is high and increasing.^17,18^ In this study we found that the antibiotic prescribing rate was very high in ICUs of Nepal. 92.85% of patients in ICU were on antibiotics, and surgical prophylaxis continued in ICU was the commonest reason for prescribing antibiotics (36%). Prolonged surgical prophylaxis, sometimes continued up to the time the patient gets discharged, is a concerning issue for all Low-and-middle income countries (LMICs).^19^ This could be an area when stewardship programs could improve antibiotic utilization.

We also aimed to see the prescribing practices of Antibiotics. We found that 67.5% of all antibiotic prescriptions were for empirical therapy. In a study done in Vietnam, 63.6% of the patients in critical care units were on empiric antibiotic therapy.^20^ Prescribing practices were poor with poor practice of defining the duration of therapy (14.37%), poor use of guidelines of therapy (27.5%), and poor utilization of biomarkers to start/change antibiotics (35.16%). Practice of sending cultures to document infection was also poor (61.1%). And cultures were sent only 34.4% of all the times when antibiotics were started or changed. Among all the cultures sent, only 26.78% had positive reports. This could also indicate that there might have been no infection in most cases.

In >50% of these, either Piperacillin/Tazobactam or Meropenem was used. This indicates most clinicians use broad-spectrum antibiotics even for surgical prophylaxis or community-acquired infections. AMR was also high with Penicillin, cephalosporins, and carbapenem groups all having resistance in > = 50% of the available culture/sensitivity reports. A good antibiotic stewardship program could decrease the misuse of these antibiotics.^21^

This study underscores the urgent need for effective antimicrobial stewardship programs in ICUs of Nepal. The high rate of antibiotic prescription, the prevalent use of broad-spectrum antibiotics even for surgical prophylaxis or community-acquired infections, and the poor adherence to guidelines and best practices highlight areas that require immediate attention. The high resistance observed against commonly used antibiotics further emphasizes the gravity of the situation.

Implementing robust antimicrobial stewardship programs could help optimize antibiotic utilization, improve patient outcomes, and ultimately combat the global threat of antimicrobial resistance. Future research should focus on developing and evaluating interventions tailored to the unique challenges faced by low and middle-income countries like Nepal. This study serves as a stepping stone towards understanding and improving antibiotic prescribing practices in ICUs of Nepal.

## Conclusion

In conclusion, there was an overprescription of broad-spectrum antibiotics for all causes of admission to ICUs. It is now crucial to establish and implement protocols/guidelines for empirical antibiotic therapy and also start to implement the surveillance of antibiotic use in ICUs of Nepal.

## Limitations

Our study had some limitations. One of the major limitations was that all ICUs in Nepal could not be involved. Being a PPS, the population captured might have been small and might not have reflected the real patient combination. Future consideration could be to design a new study with involvement of more ICUs in Nepal and design a longer-duration longitudinal study. There were also no follow-ups to know the exact duration of treatment, escalation/de-escalation based on microbiology reports, etc. Additionally, the majority of initial diagnosis of infection were made clinically, partly related to the lack of rapid diagnostics whilst the empirical antibiotic prescribing decisions were influenced by doctors’ experiences and by the level of the hospitals.

## References

[1] Mohr KI. History of antibiotics research. In: Stadler M , Dersch P , eds. How to Overcome the Antibiotic Crisis. Current Topics in Microbiology and Immunology. Cham: Springer International Publishing; 2016:237–272. 10.1007/82_2016_499 27738915

[2] Levy SB , Marshall B. Antibacterial resistance worldwide: causes, challenges and responses. Nat Med 2004;10:S122–9. 15577930 10.1038/nm1145

[3] Llor C , Bjerrum L. Antimicrobial resistance: risk associated with antibiotic overuse and initiatives to reduce the problem. Ther Adv Drug Saf 2014;5:229–241. 25436105 10.1177/2042098614554919 PMC4232501

[4] Okeke IN , Laxminarayan R , Bhutta ZA , et al. Antimicrobial resistance in developing countries. Part I: recent trends and current status. Lancet Infect Dis 2005;5:481–493. 16048717 10.1016/S1473-3099(05)70189-4

[5] Thakur S , Pokhrel N , Sharma M. Prevalence of multidrug resistant enterobacteriaceae and extended spectrum β lactamase producing escherichia coli in urinary tract infection. Res J Pharm Biol Chem Sci 2013;4:1615–1624.

[6] McEwen SA , Fedorka-Cray PJ. Antimicrobial use and resistance in animals. Clin Infect Dis 2002;34:S93–106. 11988879 10.1086/340246

[7] Curcio D. Antibiotic prescriptions in critically-ill patients: a Latin American experience. Ann Med Health Sci Res 2013;3:220. 23919194 10.4103/2141-9248.113666 PMC3728867

[8] Vincent JL. International study of the prevalence and outcomes of infection in intensive care units. JAMA 2009;302:2323. 19952319 10.1001/jama.2009.1754

[9] Vincent JL , Bihari DJ , Suter PM , et al. The prevalence of nosocomial infection in intensive care units in Europe. Results of the European prevalence of infection in intensive care (EPIC) study. EPIC international advisory committee. JAMA 1995;274:639–644. 7637145

[10] Brusselaers N , Vogelaers D , Blot S. The rising problem of antimicrobial resistance in the intensive care unit. Ann Intensive Care 2011;1:47. 22112929 10.1186/2110-5820-1-47 PMC3231873

[11] Basnyat B , Pokharel P , Dixit S , Giri S. Antibiotic use, its resistance in Nepal and recommendations for action: a situation analysis. J Nepal Health Res Counc 2015;13:102–111. 26744193

[12] Acharya KP , Wilson RT. Antimicrobial resistance in Nepal. Front Med 2019;6:105. 10.3389/fmed.2019.00105 PMC6543766 31179281

[13] Dahal RH , Chaudhary DK. Microbial infections and antimicrobial resistance in Nepal: current trends and recommendations. Open Microbiol J 2018;12:230–242. 30197696 10.2174/1874285801812010230 PMC6110072

[14] Zellweger RM , Basnyat B , Shrestha P , et al. Changing antimicrobial resistance trends in Kathmandu, Nepal: a 23-year retrospective analysis of bacteraemia. Front Med 2018;5:262. 10.3389/fmed.2018.00262 PMC6156253 30283784

[15] Hosuru Subramanya S , Bairy I , Nayak N , Padukone S , Sathian B , Gokhale S. Low rate of gut colonization by extended-spectrum β-lactamase producing enterobacteriaceae in HIV infected persons as compared to healthy individuals in Nepal. PLOS ONE 2019;14:e0212042. 30779752 10.1371/journal.pone.0212042 PMC6380550

[16] Acharya KP. National action plan for antimicrobial resistance in Nepal: possibility of translating idea into reality. Open Microbiol J 2020;14:38–39.

[17] Laxminarayan R , Matsoso P , Pant S , et al. Access to effective antimicrobials: a worldwide challenge. Lancet 2016;387:168–175. 26603918 10.1016/S0140-6736(15)00474-2

[18] Do NTT , Vu HTL , Nguyen CTK , et al. Community-based antibiotic access and use in six low-income and middle-income countries: a mixed-method approach. Lancet Glob Health 2021;9:e610–9. 33713630 10.1016/S2214-109X(21)00024-3 PMC8050200

[19] Saleem Z , Ahsan U , Haseeb A , et al. Antibiotic utilization patterns for different wound types among surgical patients: findings and implications. Antibiot 2023;12:678. 10.3390/antibiotics12040678 PMC10135394 37107040

[20] Dat VQ , Dat TT , Hieu VQ , Giang KB , Otsu S. Antibiotic use for empirical therapy in the critical care units in primary and secondary hospitals in vietnam: a multicenter cross-sectional study. Lancet Reg Health West Pac 2022;18:100306. 35024650 10.1016/j.lanwpc.2021.100306 PMC8669321

[21] Khanal S , Acharya U , Trotter AB , et al. Challenges and opportunities in the implementation of an antimicrobial stewardship program in Nepal. Antimicrob Steward Healthc Epidemiol 2023;3:e58.

